# Alpha-synuclein increases in rodent and human spinal cord injury and promotes inflammation and tissue loss

**DOI:** 10.1038/s41598-021-91116-3

**Published:** 2021-06-03

**Authors:** Andrew D. Sauerbeck, Evan Z. Goldstein, Anthony N. Alfredo, Michael Norenberg, Alexander Marcillo, Dana M. McTigue

**Affiliations:** 1 Center for Brain and Spinal Cord Repair and Department of Neuroscience, The Ohio State University, 692 Biomedical Research Tower, 460 W 12th Ave, Columbus, OH 43210 USA; 2 Departments of Pathology, Biochemistry and Molecular Biology, Neurology, Neurosurgery, and the Miami Project to Cure Paralysis, University of Miami School of Medicine, Miami, FL 33101 USA

**Keywords:** Cell death in the nervous system, Diseases of the nervous system

## Abstract

Synucleinopathies are neurodegenerative diseases in which α-synuclein protein accumulates in neurons and glia. In these diseases, α-synuclein forms dense intracellular aggregates that are disease hallmarks and actively contribute to tissue pathology. Interestingly, many pathological mechanisms, including iron accumulation and lipid peroxidation, are shared between classical synucleinopathies such as Alzheimer’s disease, Parkinson’s disease and traumatic spinal cord injury (SCI). However, to date, no studies have determined if α-synuclein accumulation occurs after human SCI. To examine this, cross-sections from injured and non-injured human spinal cords were immunolabeled for α-synuclein. This showed robust α-synuclein accumulation in profiles resembling axons and astrocytes in tissue surrounding the injury, revealing that α-synuclein markedly aggregates in traumatically injured human spinal cords. We also detected significant iron deposition in the injury site, a known catalyst for α-synuclein aggregation. Next a rodent SCI model mimicking the histological features of human SCI revealed aggregates and structurally altered monomers of α-synuclein are present after SCI. To determine if α-synuclein exacerbates SCI pathology, α-synuclein knockout mice were tested. Compared to wild type mice, α-synuclein knockout mice had significantly more spared axons and neurons and lower pro-inflammatory mediators, macrophage accumulation, and iron deposition in the injured spinal cord. Interestingly, locomotor analysis revealed that α-synuclein may be essential for dopamine-mediated hindlimb function after SCI. Collectively, the marked upregulation and long-lasting accumulation of α-synuclein and iron suggests that SCI may fit within the family of synucleinopathies and offer new therapeutic targets for promoting neuron preservation and improving function after spinal trauma.

## Introduction

α-synuclein is a small lipid binding protein abundant in CNS synaptic terminals, where it associates with pre-synaptic vesicles^1,2^. Under normal conditions, α-synuclein is involved in a variety of synaptic functions including regulation of synaptic vesicle filling and trafficking, interaction with SNARE proteins, and release of neurotransmitter^3^. Both increases and decreases in the expression of α-synuclein can dysregulate plasticity and function in the nervous system^3^, indicating that tight regulation of α-synuclein levels is critical for normal function of the nervous system. In neurodegenerative diseases such as Parkinson's disease, Alzheimer's disease, and multiple system atrophy, α-synuclein accumulates as modified protein aggregates that actively contribute to disease processes, including synaptic dysfunction, inflammation, and cell death^4–8^. In these diseases, α-synuclein aggregates form intracellular inclusion bodies^9^ or extracellular plaques with other proteins such as tau or amyloid-β. These protein modifications and aggregates lead to α-synuclein toxicity^10–14^.

In addition to damaging the cell of origin, aberrant α-synuclein can be transferred from cell to cell^15–17^, including between neurons and from neurons to astrocytes, where it is taken up by endocytosis and maintained in its aberrant conformation^18^. In this way, toxicity spreads within the CNS parenchyma. When increased in astrocytes, aberrant α-synuclein induces a pro-inflammatory phenotype leading to release of CXCL1 and TNF-α, which can directly kill neurons and oligodendrocytes^19^. This, in turn, can cause further release of intracellular proteins such as α-synuclein, which can induce a deleterious feed-forward cycle whereby astrocytes accumulate α-synuclein, release cytokines and kill more neurons, thereby releasing more α-synuclein.

Interestingly, many of the triggers for α-synuclein modification, aggregation, and release are present in animal models of spinal cord trauma, such as increased cyclic-AMP, membrane depolarization, and excess iron^20–22^. Of particular importance is iron, since it accumulates acutely after SCI and remains elevated chronically^23^. Iron drives α-synuclein expression through an iron response element (IRE) in the 5′ untranslated region (5′UTR) in the α-synuclein gene^24,25^. Binding of iron to a 5′ IRE increases the rate of translation and protein expression, suggesting that the situation is in place for enhanced α-synuclein expression in the injured spinal cord. If present after SCI, pathological α-synuclein would be in position to exacerbate tissue pathology through multiple mechanisms, such as elevated reactive oxygen species production^26^, mitochondrial impairment^27^, and synaptic dysregulation^14^. Indeed, recent work using non-traumatic models of spinal cord injury (SCI) in lampreys and rabbits suggests that α-synuclein could contribute to tissue pathology and lack of regeneration^28–30^.

In the current study, we first asked if iron and/or α-synuclein accumulate after human SCI. Results show both robust α-synuclein and iron accumulation in acute human SCI tissue, with prominent staining of α-synuclein in damaged white matter and astrocyte-like profiles. Next, protein from rat traumatized spinal cords was probed for expression of monomeric α-synuclein, which revealed both truncated and elevated molecular weight isoforms from acute to chronic post-injury times. Last, knockout mice were used in a model of clinically relevant traumatic SCI to test the hypothesis that α-synuclein contributes to post-SCI tissue pathology. Results showed deletion of α-synuclein significantly increased axon and neuron sparing and reduced inflammatory markers in mouse SCI tissue. Evaluation of locomotor recovery revealed an intriguing interaction between α-synuclein and dopamine that appears to be necessary for optimal motor recovery. Given the well-established link between aberrant α-synuclein accumulation and cell death^14,31,32^, these results identify a new pathological mechanism in the injured rodent and human spinal cord which may present a therapeutic target for reducing tissue pathology and improving recovery from SCI.

## Results

### α-synuclein and iron deposition are present in the injured human spinal cord

**Figure 1:**
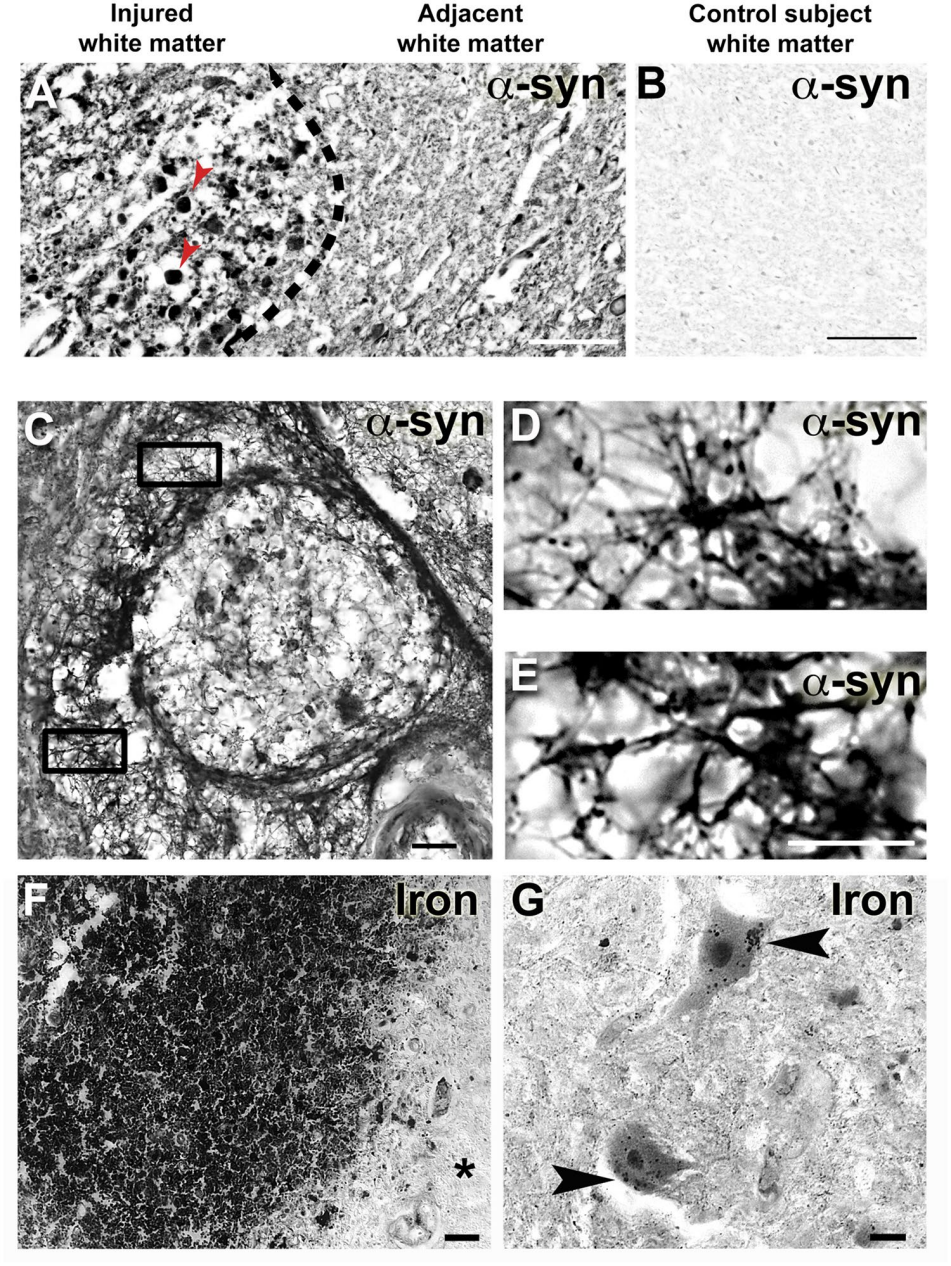
α-synuclein and iron increase in the human spinal cord after SCI. (**A**) LB509 + α-synuclein was present in injured white matter at 12d post-injury, and localized to large aggregates resembling axons within the injured white matter (red arrowheads). (**B**) Pathological α-synuclein was not present in white matter from non-injured human spinal cords. (**C**) α-synuclein was also present after human SCI in cells resembling astrocytes around the lesion site at 12d post-injury. (**D,E**) High magnification of cells boxed in panel (**C**). (**F**) Human SCI tissue stained for ferric iron using Perls stain shows dense iron accumulation within the injured region; adjacent spared tissue (*) had no visible iron stain. (**G**) Section of human SCI showing spared neurons close to lesioned tissue appear to have iron + inclusions in their cytoplasm (arrowhead). Scale bars: 50 µm in (**A**) and (**B**); 20 µm in (**C**,**E,G**)**;** 400 µm in (**F**).

To determine if α-synuclein accumulates after human SCI, injured spinal cord tissue sections were immunolabeled with the LB509 antibody, raised against human α-synuclein from Lewy bodies. To confirm the pathological nature of the α-synuclein, human SCI sections were treated with proteinase-k, which degrades normal but not pathological α-synuclein^33,34^. This revealed no labeling in uninjured human SCI sections, as expected. In contrast, abundant LB509 + α-synuclein profiles were present within and around lesions in 11-12d post-injury human SCI tissue (Fig. 1A–E). No-primary controls on injured human tissue confirmed a lack of labeling (not shown). In total, LB509 immunoreactivity was absent in five uninjured human spinal cords (Fig. 1B) while all four human SCI samples contained abundant α-synuclein-positive profiles with astrocyte morphology, and three of four contained large α-synuclein aggregates in the white matter, suggesting a potential involvement of axons (Table 1). Thus, spinal cord trauma in humans results in acute and marked upregulation of α-synuclein.

**Table 1:** Characteristics of human spinal cord injury and control subjects. There were no differences in age or gender between injured (Ages: 19–90, Gender: 3 males and 1 female) and control (Ages: 20–80, Gender: 4 males and 1 female) subjects. Spinal cord sections from three of four injured subjects had axon profiles with immunoreactivity for aggregate α-synuclein (LB 509), and all four injured subjects had immunolabeling for α-synuclein in astrocyte-like profiles. All four also had robust iron accumulation in lesioned areas. Control spinal cords showed no immunoreactivity for LB509 + α-synuclein profiles or iron deposition. MVA = motor vehicle accident, GSW = gunshot wound. D.P.I. = days post-injury until death.

Spinal cord injury subjects				
Cause of death	Injury level	D.P.I	White matter	Astrocytes
Fall	C1	11	–	+
MVA	C6	12	+	+
GSW	L1	12	+	+
MVA	C4	12	+	+

Control subjects				
Cause of death	Injury level	D.P.I	White matter	Astrocytes
MVA	None	N.A	–	–
MVA	None	N.A	–	–
MVA	None	N.A	–	–
GSW	None	N.A	–	–
Unknown	None	N.A	–	–
		P-value	0.0476	0.0079
			Fisher exact	Fisher exact
			Two-tailed	Two-tailed

A well-known instigator of α-synuclein expression and modification is excess iron. Our prior work showed robust accumulation and deposition of iron in traumatized rodent spinal cords^23^,1,

### Modified α-synuclein monomers are present chronically in the injured rat spinal cord

**Figure 2:**
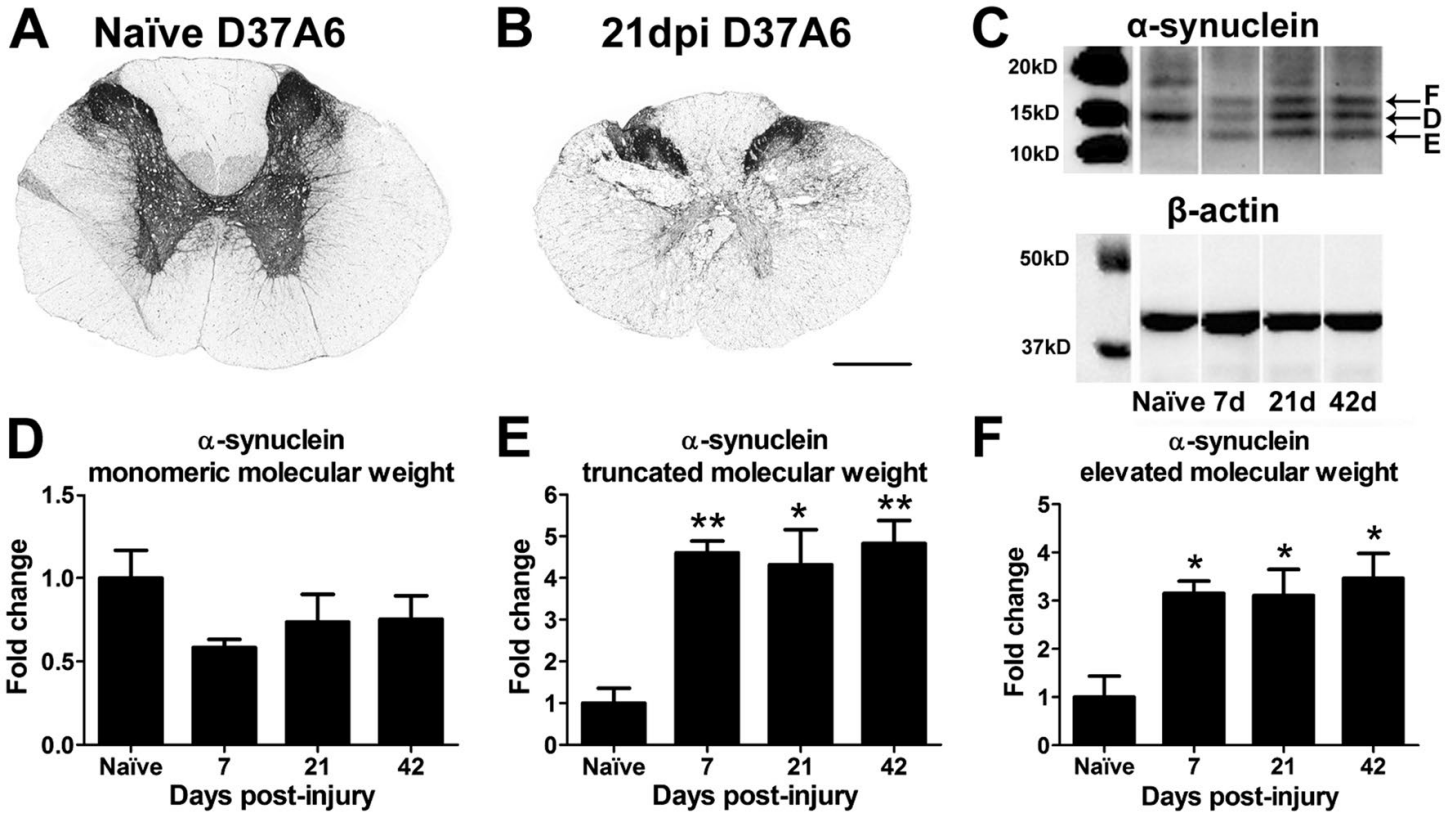
Aberrant forms of native α-synuclein are present after spinal cord injury in rats. (**A**) Antibody labeling for native monomeric α-synuclein (D37A6) revealed the expected immunoreactivity in the grey matter in uninjured rat spinal cords. (**B**) Following SCI, native α-synuclein was lost in areas of damaged grey matter. Image taken from Sect. 21d post-injury (dpi). (**C**) Representative western blot of monomeric α-synuclein stained with the D37A6 antibody illustrating protein modifications after SCI. Protein was isolated from naïve, 7d, 21d and 42d post-injury. White dividing lines indicate borders of cropped representative fields from the same western blot. (**D**) Quantification of normal molecular weight isoform of α-synuclein protein showed a non-significant reduction after SCI. (**E,F**) Truncated and larger molecular weight isoforms of α-synuclein were significantly increased 7d–42d after spinal cord injury compared to uninjured controls. n = 3–4/group. Two-way repeated measures ANOVA with Bonferroni post-hoc test. *p < 0.05, **p < 0.01.

Pathological environments lead to α-synuclein protein modifications, including truncation, fibrillization, nitration and oxidation. To examine modification of α-synuclein after experimental SCI, rats received a T8 moderate mid-thoracic contusive SCI. Cross-sections were immunolabeled with the D37A6 antibody generated against monomeric (“normal”) isoform of the protein. The labeling exhibited the expected pattern of positive signal in the gray matter both before and 21d after SCI (Fig. 2A,B). Western blot analysis from naïve and 1w–6w post-injury revealed a non-significant 40% reduction in the normal monomeric form of α-synuclein (~ 14kD) after SCI (Fig. 2C,D). However, monomeric α-synuclein variants with altered molecular weights increased significantly 1w–6w post-injury (Fig. 2C,E,F). Thus, aberrant forms of α-synuclein are present chronically within the injured rodent spinal cord.

### α-synuclein knockout improved neuron and axon sparing and reduced iron deposition after SCI in mice

**Figure 3:**
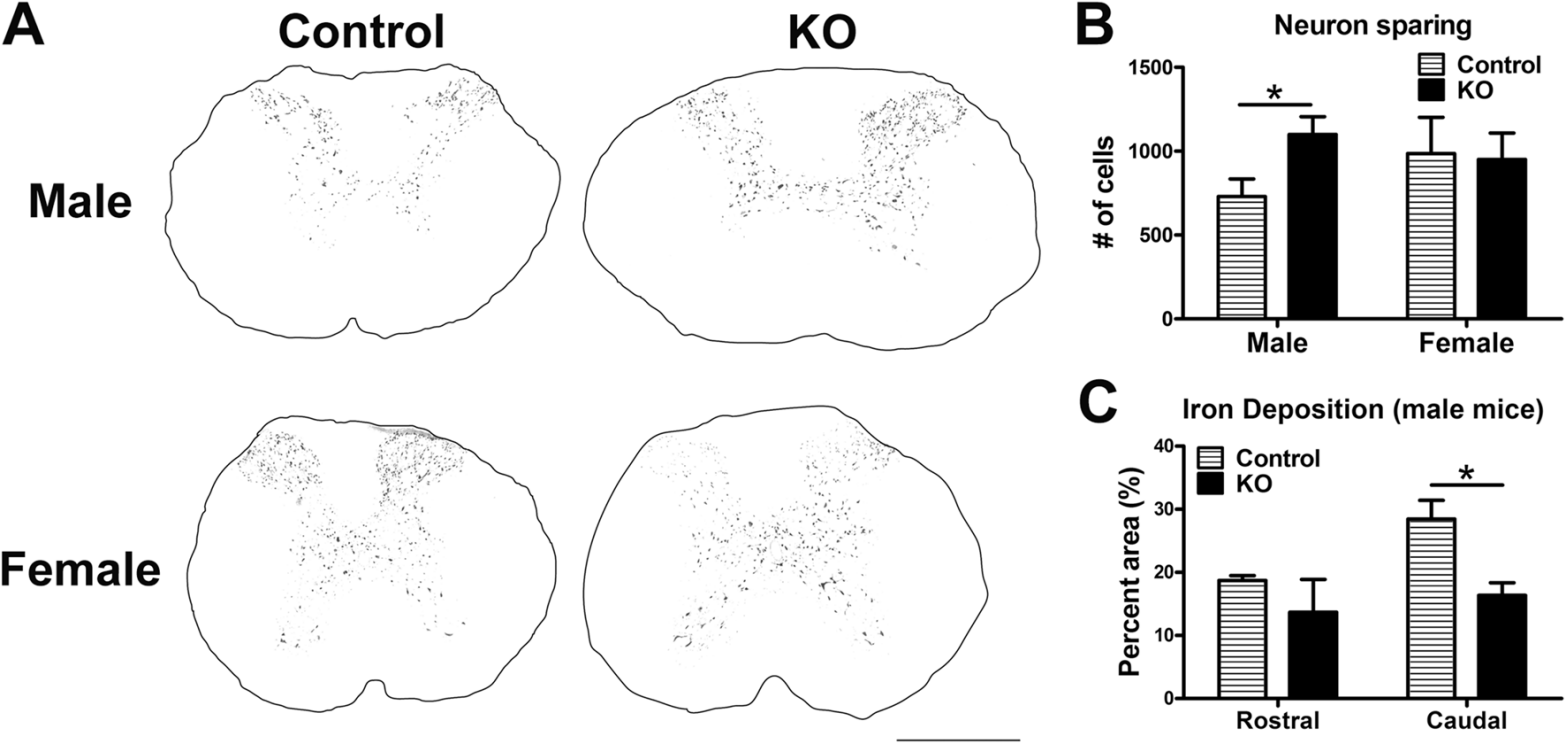
Neuron loss and iron deposition are reduced after SCI in male α-synuclein knockout mice. (**A**) Representative images of NeuN stained tissue at 600 µm rostral to the injury epicenter. (**B**) Quantification of NeuN positive neurons ± 600 µm of the lesion epicenter. Male α-synuclein knockout mice had significantly more spared neurons in tissue ± 600 µm of the lesion epicenter compared to controls. Neuron sparing was not different in female mice. n = 4–9/group. Two-tailed t-test *p < 0.05. (**C**) Quantification of intraspinal iron accumulation 600 µm rostral or caudal to the injury epicenter in male mice. α-synuclein knockout mice had significantly lower intraspinal iron caudal to the injury epicenter compared to control mice. n = 4–7/group. Two-way repeated measures ANOVA with Bonferroni post-hoc test. *p < 0.05*.* Scale bar: 500 µm.

**Figure 4:**
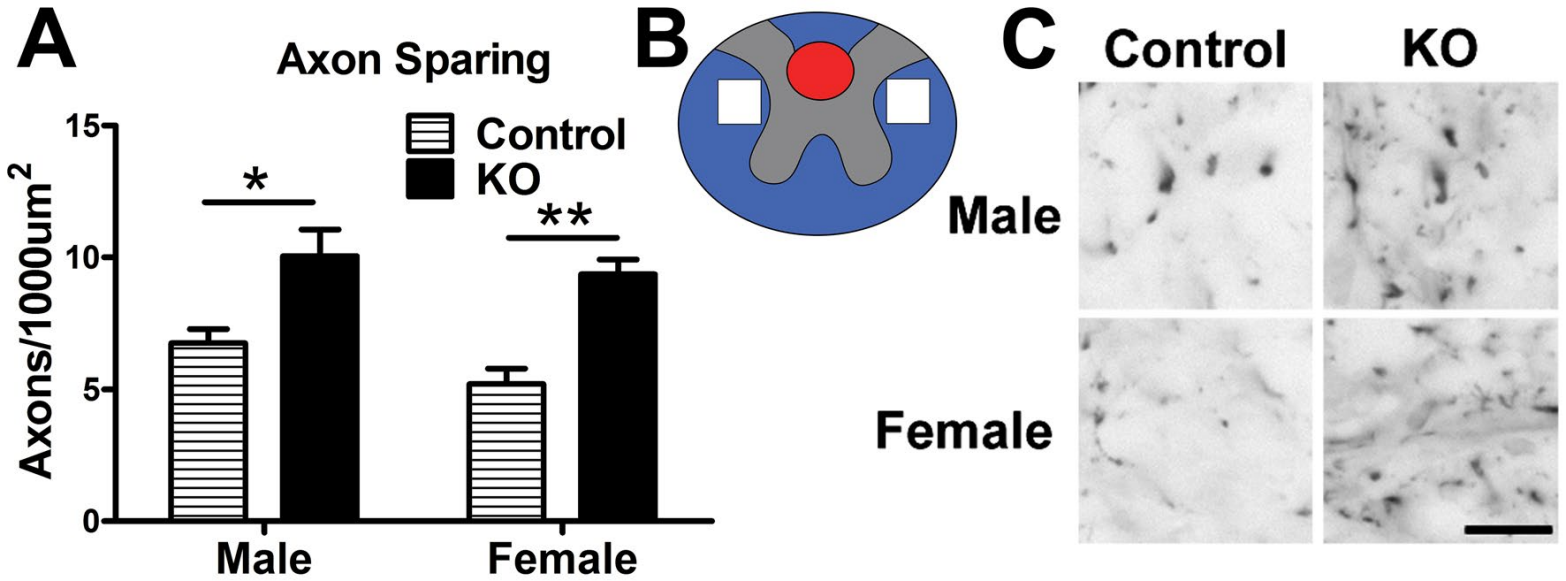
Axon sparing is increased in α-synuclein knockout mice after SCI. (**A**) Quantification of neurofilament-heavy positive axons 600 µm rostral of the lesion epicenter. Both male and female α-synuclein mice had significantly increased axon sparing after SCI. (**B**) Schematic representation of regions of axon quantification. White boxes represent quantified areas. Red area represents region of frankly damaged tissue 600 µm rostral of the lesion epicenter. (**C**) Representative images of neurofilament positive axons from sampled regions. n = 3–4/group. Two-way repeated measures ANOVA with Bonferroni post-hoc test. *p < 0.05, **p < 0.01. Scale bar equals 12.5 µm.

To determine if α-synuclein aggregation/modification is simply a bystander effect of tissue injury or if it actively contributes to tissue pathology, male and female mice with a homozygous deletion of α-synuclein (α-synuclein KO) or littermate controls received a T9 moderate mid-thoracic contusive SCI and survived to 7d or 21dpi. Immunohistochemical labeling of α-synuclein using the D37A6 antibody verified a complete lack of staining in KO mice but showed the expected gray matter labeling in control mice before and after SCI (data not shown). At 21 dpi, tissue sparing was analyzed in spinal cord cross-sections within 600 µm of the epicenter. Male (but not female) α-synuclein KO mice had significantly more spared neurons than controls (Fig. 3A,B). Iron deposition was also significantly reduced caudal to the epicenter in male α-synuclein KO mice at 7dpi, suggesting reduced pathology and cell death in knockout mice (Fig. 3C). Tissue sparing at the epicenter was not different, but both male and female KO mouse spinal cords had significantly more spared axons at 21dpi in tissue at the poles of the lesions (Fig. 4A–C). Collectively these results indicate that α-synuclein exacerbates tissue pathology in the injured spinal cord.

Despite increased distal axon sparing, the overall spared white matter area based on EC stain throughout the rostral/caudal extent of the lesion was not different between genotypes (Supp. Figure 1), suggesting that a higher number of axons per white matter area were present in α-synuclein KO mice. Matching the preservation of white matter area, the number of oligodendrocytes also was not different between the groups (Supp. Figure 1). Given the presence of α-synuclein profiles resembling astrocytes after human SCI (Fig. 1), GFAP immunoreactivity was compared in the region of increased axon sparing between KO and control mice, which also revealed no significant difference between α-synuclein KO and control mice of either sex (Supp. Figure 1).

### α-synuclein knockout reduced inflammatory markers after SCI in mice

**Figure 5:**
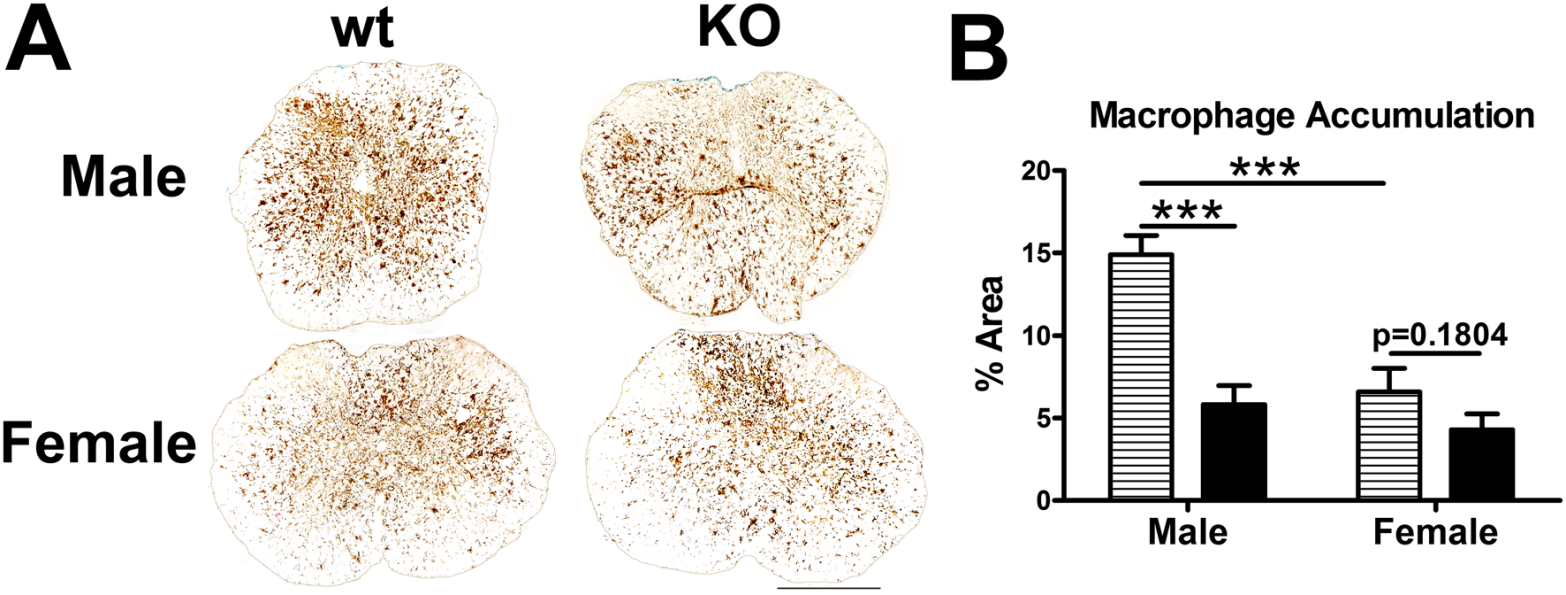
α-synuclein knockout reduces intraspinal macrophage accumulation after SCI. (**A**) Representative images of Cd11b-positive macrophages 600 µm rostral to the injury epicenter. (**B**) Male KO mice had significantly reduced macrophage accumulation (Mac-1 immunoreactivity) compared to controls across the lesion extent (± 600 µm from epicenter). Macrophage accumulation was not different in female KO and control mice; however female control mice had significantly reduced overall Mac-1 immunoreactivity compared to male controls. n = 4–5/group. Two-way repeated measures ANOVA with Bonferroni post-hoc test. ***p < 0.001. Scale bar equals 500 µm.

Improved axon and neuron sparing suggests the overall pathology was reduced by α-synuclein knockout. To determine if this was associated with a difference in inflammatory-related indices, total cross-sectional microglia/macrophage accumulation was compared using the area of Cd11b immunoreactivity ± 600 µm from the epicenter (the region of increased axon and neuron sparing). In male α-synuclein KO mice, macrophage accumulation was significantly reduced compared to wild type controls (Fig. 5A,B). Female KO mice had a trend towards a reduction compared to controls (Fig. 5A,B). Female control mice also had significantly reduced immunoreactivity for activated macrophages compared to male control mice (Fig. 5A,B).

**Figure 6:**
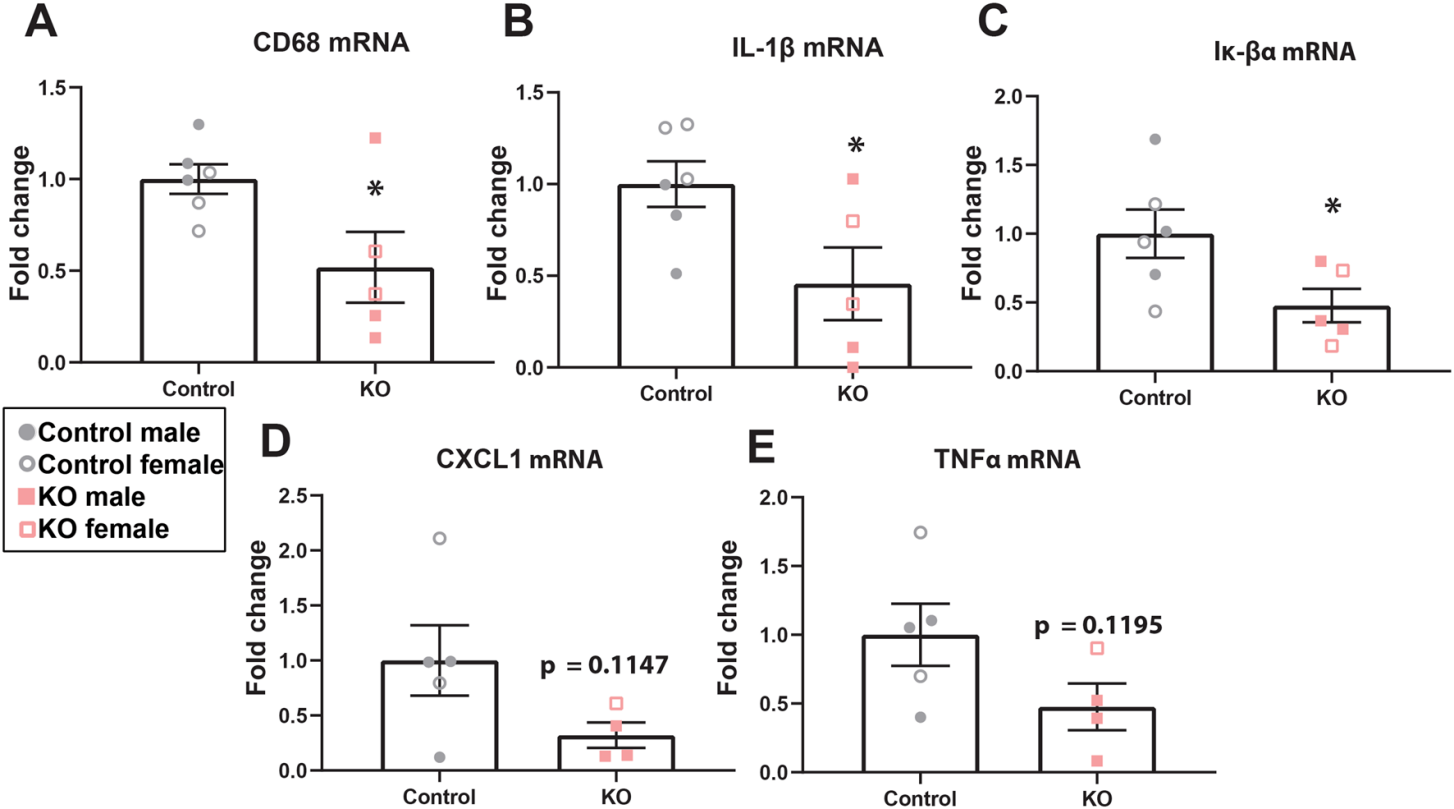
α-synuclein knockout mice have altered inflammatory markers after SCI. (**A–C**) Real-time PCR of RNA collected from the injury site at 21d post-injury showed a significant reduction in CD68, IL-1β and Iκβα RNA in KO mice compared to controls. (**D–E**) There was a trend towards a decrease in RNA levels of CXCL1 and TNFα. n = 4–6/group. Two-tailed t-test. *p < 0.05.

Reduced inflammatory mediators at 21dpi was confirmed using real-time PCR, which revealed significantly lower CD68 mRNA and interleukin-1β mRNA in α-synuclein KO mice spinal cords (Fig. 6A,B). Next we examined I_K_βα mRNA, which is a negative feedback regulator of NF_K_B signaling and is increased when NF_K_B is activated, therefore increased I_K_βα is commonly used as an indicator of NF_K_B activation (Quan et al*.,* 1997, LaFlamme et al*.,* 1999). In line with lower inflammation in α-synuclein KO spinal cords, I_K_βα mRNA was significantly lower in α-synuclein KO spinal cords compared to controls, indicating reduced NF_K_B activation. Though not statistically significant, there were also trends towards decreased CXCL1 and TNFα mRNA in KO mice (Fig. 6D,E).

### α-synuclein KO impairs locomotor recovery from SCI, likely by exacerbating dopamine signaling dysfunction in these mice

**Figure 7:**
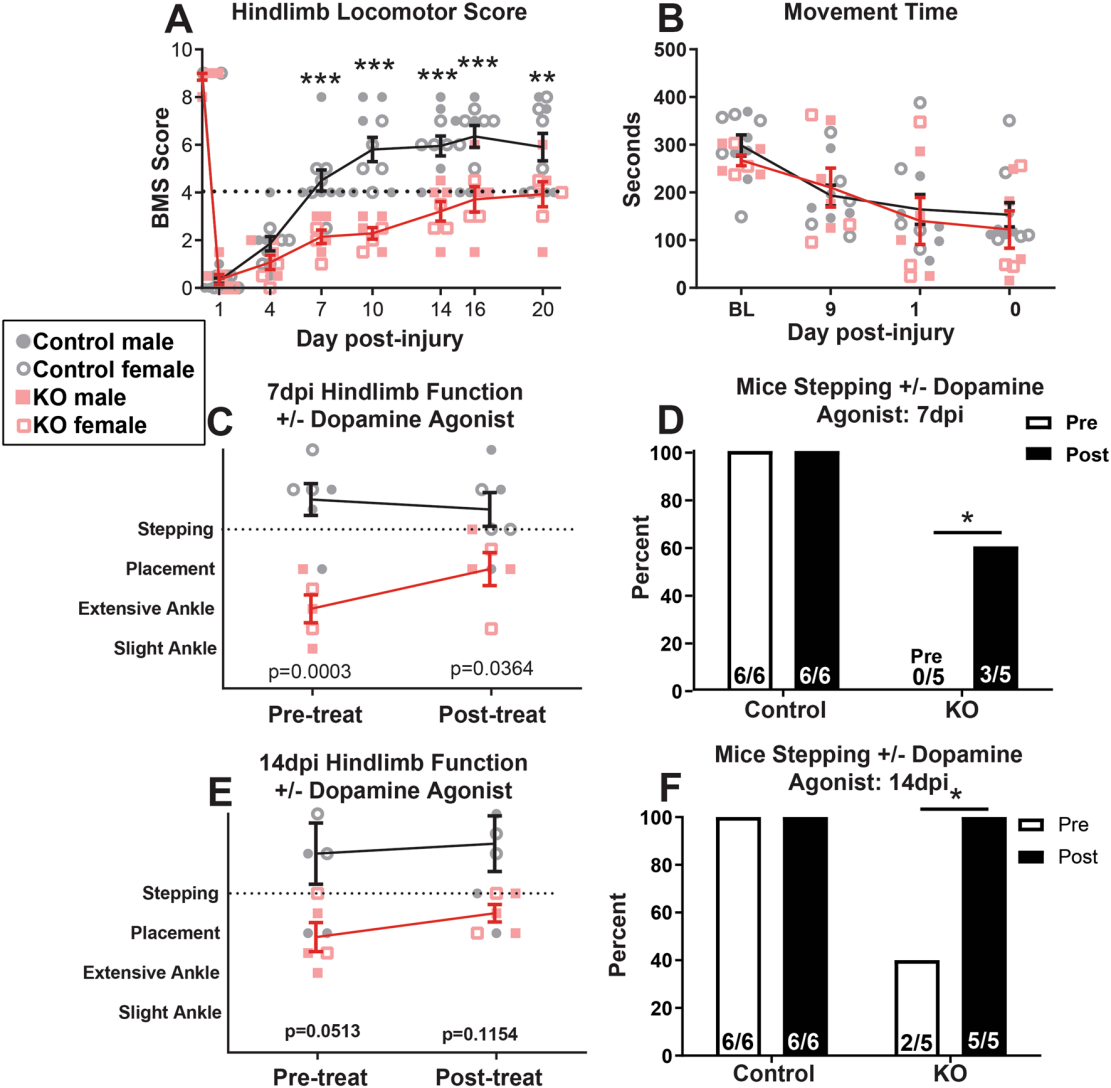
Mice lacking α-synuclein had worse locomotor recovery after spinal cord injury, likely due to dopamine deficiency. (**A**) Hindlimb function assessed with the BMS rating scale revealed KO mice had significantly worse locomotor recovery, including a marked reduction in stepping ability, compared to controls 7–20 days post-injury (dpi). Horizontal line indicates a BMS score of 4, which is the threshold for achieving plantar stepping. (**B**) Movement time was not different between genotypes at any time post-injury. (**C**,**D**) Treatment with the dopamine agonist SKF-81297 at 7dpi transiently reversed the stepping impairment in the KO mice and improved BMS scores to control levels (**C**). The number of KO mice stepping increased from zero before treatment to three 15 min after treatment (**D**). (**E**,**F**) Treatment with the dopamine agonist at 14dpi slightly increased locomotor function in KO mice (**E**) and significantly increased the number of KO mice stepping 15 min after treatment (**F**). n = 5–6/group. (**A**–**C**,**E**): Two-way repeated measures ANOVA with Bonferroni post-hoc test. D, F: Chi-squared test. *p < 0.05, ***p < 0.001.

To determine if loss of α-synuclein altered functional recovery after SCI, open-field locomotion was evaluated before and after SCI using the BMS open field locomotor scale^35^. Prior to SCI, α-synuclein KO and control mice both had normal locomotor function (BMS score = 9; Fig. 7A). Surprisingly, after SCI, locomotor recovery was significantly worse in α-synuclein KO mice (of both sexes) compared to controls, as early as 7dpi (Fig. 7A). While control SCI mice regained the ability to consistently step with some forelimb/hindlimb coordination (BMS score = 6), α-synuclein KO mice could only occasionally step at best and never regained forelimb/hindlimb coordination (BMS score ≤ 4). Despite worse locomotor function, total locomotor activity as assessed by movement time in activity boxes was not different between control and α-synuclein KO mice post-injury, showing that general activity levels were not reduced in KO mice (Fig. 7B).

The reduced locomotor recovery in KO mice after SCI suggests that the lack of α-synuclein resulted in a deficit that overrode the neuroprotective effect of α-synuclein removal. Previous work showed that this strain of α-synuclein KO mice have normal brain architecture and typical dopamine uptake and discharge; however, they do exhibit impaired dopaminergic function, which reduces dopamine-dependent locomotor response after amphetamine treatment^7^. To investigate if reduced dopaminergic signaling contributed to impaired hindlimb function after SCI in α-synuclein KO mice, BMS scores were collected in mice before and 15 min after boosting dopamine signaling by treatment with the D1/5R agonist SKF-81297 at 7d and 14 dpi.

As shown in Fig. 7A, at 7 dpi no KO mice could perform spontaneous plantar stepping, which is a BMS score of 4; most displayed only extensive ankle movement at best (BMS = 2; Fig. 7C). Within 15 min of dopamine agonist treatment, however, all KO mice transiently gained the ability to plantar place their hindlimbs, and 3/5 KO mice could plantar step (Fig. 7C,D). In contrast, dopamine agonist had no effect on control mouse locomotor function (Fig. 7C).

Similar results were obtained at 14dpi when the dopamine agonist again increased stepping ability in KO mice. Dopamine agonist increased overall BMS scores from ~ 3 (plantar placement) to 4 (occasional plantar steps) (Fig. 7E), and the number of mice that could spontaneously plantar step increased significantly from only two KO mice to all KO mice (5/5) (Fig. 7F). As before, the dopamine agonist had no effect on stepping ability or BMS scores in control mice (Fig. 7E,F). Based on activity box measurements, dopamine agonist treatment did not alter the amount of time spent moving in either genotype, indicating that the dopamine agonist did not increase stepping by promoting overall locomotor activity. Collectively these results suggest that dopamine dysfunction exacerbates locomotor deficits after SCI in mice completely lacking α-synuclein.

## Discussion

α-synuclein has a well-established role in neurodegenerative diseases or synucleinopathies^4–6,36,37^. Prior to the current study, the contributions of α-synuclein to pathology after traumatic contusive SCI received limited attention. This work shows that reducing α-synuclein significantly increased axon sparing and reduced measures of inflammation after SCI.

Importantly, LB509-positive proteinase K-resistant α-synuclein profiles were detected in 2w post-injury spinal cords from human subjects. Of the injured cases examined, all four contained α-synuclein-positive profiles resembling astrocytes and three contained robust α-synuclein aggregates in injured white matter. Among these cases, the two suffering motor vehicle accidents had the most robust α-synuclein expression, suggesting trauma may exacerbate accumulation of this toxic protein. Resistance of α-synuclein to proteinase K degradation in the human samples tested here indicates the post-SCI α-synuclein is pathological^38^ since this enzyme degrades non-pathological α-synuclein.

What would cause α-synuclein to form pathological aggregates after SCI? One well-characterized trigger abundant after SCI is iron, as both ferric and ferrous iron induce α-synuclein to form toxic aggregations^39^. It is well known that intraspinal hemorrhage leads to robust iron accumulation, and that excess iron is maintained chronically after SCI in rodents^23^. This sets the stage for prolonged α-synuclein aggregation in the injured spinal cord. Further, the α-synuclein gene contains a putative iron-response element (IRE) meaning that iron can not only cause aggregation of pre-existing α-synuclein, but that it could also drive translation of new intraspinal α-synuclein^24^. Interestingly, α-synuclein can function as a ferri-reductase and thereby increase pro-oxidant ferrous iron, which could drive a feedback loop producing more α-synuclein and thereby exacerbate cell injury^40^. Notably, we show robust ferric iron accumulation in human SCI lesions and in neurons close to the injury site. Therefore, iron is in the right place and time to play a role in toxic α-synuclein accumulation after human SCI. Though the present studies focused specifically on α-synuclein, both β- and γ-synuclein accumulation have been observed in humans with brain iron accumulation suggesting that similar mechanism may be involved with other synucleins^41^. Given alterations in γ-synuclein are observed after SCI^30^ and TBI^42^, it is possible that these other synucleins change in parallel to our observed changes in α-synuclein after SCI. Since global knockout of α-, β-, and γ-synuclein are not lethal, it is plausible that there is functional overlap of these proteins both in normal conditions and after SCI. Future studies should explore the role of all synucleins after SCI as this could explain some of the results in the current studies, such as neuron sparing without improvements in motor function. If the functional overlap the synuclein isoforms is meaningful and all synucleins have a pathologic role after SCI, then a broader, non-subtype specific, therapeutic approach would likely yield superior histological outcomes.

Acidic environments, such as the spinal contusion site, also drive α-synuclein aggregation, as does oxidation or nitration of α-synuclein^39,43^. Indeed, prior studies revealed that 4-hydroxy-2-nonenal (HNE), a potent oxidant present after SCI, induces the production of α-synuclein oligomers that are cytotoxic to neurons^44,45^. Oxidants such as HNE also induce post-translational modifications and truncation of the α-synuclein protein, such as that seen here after rat SCI; these modifications facilitate its aggregation and toxicity^45–47^. Since α-synuclein is ubiquitously expressed by neurons and anterogradely transported in axons, it will be present in and around the SCI site at the time of trauma and would be vulnerable to the pro-aggregating and protein modifying conditions of the injured milieu. Accordingly, we noted a significant increase in modified isoforms of α-synuclein monomers with lower and higher molecular weight variants present in the injured spinal cord for at least 6 weeks after SCI. Elucidating the specific structural changes in the α-synuclein monomers will be useful in understanding the potential mechanisms leading to these changes. Such modifications play a direct role in the development of neurodegenerative disease and synucleinopathies (for review, see^46^) and could similarly exacerbate pathology and impair recovery after spinal trauma. A limitation of the present studies is that only female rats were used for the molecular analysis of α-synuclein monomers. Though our findings of elevated and truncated molecular weight α-synuclein monomers show changes that are occurring after contusive SCI in female rats, the fact that male mice showed the most robust effects of α-synuclein KO while female mice showed limited or mixed effects indicates there may be a disconnect between the modified monomers observed in rats and the histological and behavioral changes observed in mice. As such, future studies are needed to elucidate both gender and species differences in the role of structurally modified α-synuclein after SCI in mice.

There are multiple mechanisms through which aberrant α-synuclein could induce post-SCI tissue pathology and dysfunction. First, accumulation of α-synuclein impairs axon transport and causes axonopathy^48,49^. Axonal α-synuclein aggregates can also prevent axon regeneration^50^. Work using a lamprey model of spinal transection revealed that neurons classified as “bad regenerators” had chronic upregulation of synuclein after axotomy, in contrast to successfully regenerating neurons that had no change in α-synuclein levels^28,30^. Thus, axons transected by the injury that accumulate α-synuclein may be resistant to spontaneous or therapeutically-induced regeneration cues. Interestingly, in a rat hemi-section SCI model, α-synuclein gene expression was reduced in the ipsilateral spinal cord^51^, suggesting differences in pathological mechanisms may exist between cut vs. traumatic spinal cord injuries. In addition to intracellular accumulation, α-synuclein can be released extracellularly, likely by calcium-dependent exosomes^52^, and then taken up by neighboring cells. Thus, through cell-to-cell transmission, α-synuclein toxicity can move from one cell to another and could theoretically spread throughout the injured spinal parenchyma. Indeed, in vitro evidence showed that aberrant α-synuclein can be endocytosed by axons, where it converts endogenous soluble α-synuclein into insoluble aggregates; over time these aggregates propagate to the cell bodies and lead to neuron death^14^. Here we show that male and female α-synuclein KO mice had greater axon sparing after SCI compared to controls, suggesting that α-synuclein contributes to axon pathology. Knockout of α-synuclein also increased neuron sparing but only in male mice. The lack of effect in females may be related to differences in injuries between male and female mice, such as female control mice having reduced inflammation compared to male control mice. These sex dependent differences in histological outcomes for male and female mice should be explored in detail as it will likely shed light on other mechanisms regulating α-synuclein after SCI. It is plausible that either the presence or role of aberrant α-synuclein after SCI differs between males and females and if true could help explain the sex dependent differences observed in the current studies. The protective effects of α-synuclein KO likely were mediated by reducing its known pathological actions, such as α-synuclein-induced rise in intracellular free iron and/or enhanced calcium influx into the cells, both of which can drive formation of the powerful pro-oxidants H_2_O_2_ and reactive oxygen species^53–55^. Supporting the potential role of α-synuclein-induced iron dysregulation, KO mice had reduced intraspinal iron accumulation compared to controls, indicative of less tissue pathology despite comparable injury biomechanics.

In addition to neurons, astrocytes readily take up extracellular α-synuclein, both in vitro and in vivo^18^. Chronic post-SCI uptake and/or expression by astrocytes could have deleterious consequences since aberrant α-synuclein induces a pro-inflammatory phenotype in astrocytes and promotes robust release of molecules such as TNFα and CXCL1^18^. Since astrocytes play a pivotal role in post-SCI inflammation^56,57^, excess α-synuclein could bias them toward a deleterious phenotype long-term. Indeed, astrocytes forced to over-express α-synuclein kill spinal motor neurons and induce paralysis in animals^19^. Thus, the persistence of α-synuclein-positive astrocytes after SCI in otherwise intact tissue may contribute to prolonged inflammatory activation and could limit spontaneous or therapeutically-induced repair^58^.

Given the well-established link between α-synuclein aggregation and tissue pathology, we hypothesized that removal of α-synuclein would promote tissue sparing after SCI. Indeed, neuron and axon preservation were enhanced and indicators of inflammation were reduced in post-SCI α-synuclein KO mice, with the most consistent effect across endpoints being observed in male mice. Similarly, lenti-viral reduction of α-synuclein showed improved tissue sparing and reduced inflammation even in more mild cases of contusive SCI^59,60^. Despite the observed histological neuroprotection in our study, α-synuclein KO mice had worse hindlimb locomotor recovery compared to controls after SCI. This is an important finding as it suggests that completely and permanently reducing α-synuclein may be harmful to a patient. To fully understand the risk–benefit relationship of targeting α-synuclein after SCI, employing a therapeutically tractable intervention such a temporary administration of an α-synuclein lowering drug post-injury, rather than the global knockout, is needed. In contrast to our present studies, in a SCI model where animals spontaneously regain the ability to step, α-synuclein reduction showed a modest improvement in hindlimb function^59,60^. This suggests that α-synuclein reduction can have direct functional benefits but they may be dependent on injury severity or specific aspects of motor function^59,60^. The best anatomical correlate to locomotor recovery after thoracic SCI is the amount of white matter spared at the epicenter (Basso et al., 1995). However, overall white matter sparing was identical in KO mice and controls in our study. If anything, improved neuron and axon sparing in KO mice would predict better functional recovery, although increased thoracic neurons may have little direct effect on hindlimb motor control. Though a limitation of our behavioral studies was not being powered to statistically separate and compare male and female mice, it is intriguing that general distribution of male and female data points overlap across the functional tests. Unlike the observed sex dependent differences in our histological outcomes, the similarities between male and female α-synuclein KO mice behaviorally suggests that the functional outcomes are more similar between sexes than the histological outcomes.

Based on the similar lesion size and improved neuroprotection in KO mice, we hypothesized that neurochemical differences between α-synuclein KO and control mice drove the locomotor deficits. A system that plays an important role in locomotor control is the descending dopamine pathway from the hypothalamus, which projects through the thoracic cord to the lumbar locomotor region^61,62^. α-synuclein is known to play a particularly important role in dopamine release^7,63,64^ and, accordingly, the KO mice used here have altered dopamine release/re-uptake kinetics which impairs amphetamine-induced locomotor activity^7^. Thus, injury to other descending motor tracts after SCI may have unmasked and exacerbated the dysfunctional dopamine system, which would limit motor recovery to a greater extent than in control mice. We tested this by “adding back” dopamine to KO and control mice. That is, post-SCI mice were given a dopamine agonist to determine if enhancing dopamine signaling would temporarily improve hindlimb function in α-synuclein KO mice. This was the case, as KO mice unable to step before treatment gained the ability to produce weight-supported plantar steps while the drug was on board. This made their hindlimb function comparable to wild type mice, whose hindlimb function was unaffected by the dopamine agonist. These results are consistent with work showing that intraspinal dopamine potentiates weight bearing stepping^62^, and are further supported by studies in which mice given spinal transections, which eliminate all descending dopamine axons, had improved hindlimb function when given the same dopamine agonist used here^65,66^. Thus, impaired locomotor recovery after SCI in α-synuclein KO mice may be an artifact of less-than-optimal functioning of their dopamine system and not due to reduced intraspinal α-synuclein. This does, however, highlight the importance of dopamine signaling in hindlimb locomotor control after SCI. Furthermore, considering that both male and female mice responded similarly to the administration of SKF-81297 with improved hindlimb performance suggests that similar pathways are activated to improve performance across sexes. This indicates a degree of similarity between males and females following SCI which is not as apparent in the histological endpoints, which are more variable between genders. Though the lack of a behavioral benefit in α-synuclein KO mice pre- SKF-81297 is disappointing, it is possible that in a clinically relevant setting, temporarily reducing α-synuclein could improve histological outcome which in turn could improve recovery when α-synuclein levels return to baseline. Future studies using a transient therapeutic approach to reduce α-synuclein during the acute phase post-injury will be needed to address this potential scenario. Our findings with SKF-81297 support the hypothesis that a transient reduction of α-synuclein may not impair behavioral since the structural pathways needed to engage hindlimb function are clearly still present but were rendered non-functional due to the global persistent absence of α-synuclein.

Collectively, this work shows robust α-synuclein aggregation and iron accumulation in human SCI tissue for the first time, raising the possibility that aberrant α-synuclein enhances tissue pathology in patients suffering from SCI. Knockout of α-synuclein in mice was neuroprotective and reduced markers of inflammation, suggesting α-synuclein enhances post-SCI tissue pathology. Thus, identifying and targeting the mechanisms that convert naive α-synuclein to aggregates of insoluble toxic protein species may lead to new clinically important therapies to improve recovery from SCI.

## Material and methods

### Animals and spinal cord injury

Spinal cord contusions were performed using standardized protocols as previously described^23,67–70^. All procedures conformed to the ARRIVE guidelines and were approved by The Ohio State University IACUC. All methods were carried out in accordance with relevant guidelines and regulations. For rat studies, n = 3–4 adult female Sprague–Dawley rats were anesthetized as above and received a dorsal laminectomy at the T8 vertebral level. They then received a moderate spinal contusion using a pre-set for or 200 kD. Postsurgical care for mice and rats included 5 days of antibiotics (gentamicin, 5 mg/kg) and saline to maintain hydration, and twice-a-day manual bladder expression until spontaneous voiding returned. Mice were exsanguinated at 21d post-injury (n = 3–5/group). Rats were exsanguinated at 7d, 21d or 42dpi (n = 3–4/group); naïve uninjured rats were used as controls (n = 3). For knockout mouse studies, male and female homozygous α-synuclein knockout mice (Jackson Labs B6;129X1-Sncatm1Rosl/J) or controls generated by crossing C57BL/6 J and 129X1/SvJ founders (Jackson labs) were used. Mice were anesthetized with ketamine (80 mg/kg, i.p) and xylazine (10 mg/kg, i.p.), and a dorsal laminectomy was performed at the T9 vertebral level. Mice then received a moderate spinal contusion injury using the Infinite Horizons device (Precision Systems and Instrumentation) with a preset force of 75 kD. The muscles overlying the spinal cord were sutured and the skin was closed with surgical clips. Animals were given 2 ml of saline and placed into warm recovery cages immediately following the injury. During the studies one mouse was excluded at the time of injury for an impact that hit bone and three mice died following injury.

### Human tissue samples

Postmortem human spinal cord samples were obtained in accordance with relevant guidelines and regulations. Informed consent was obtained from a legally authorized representative. The research protocol was approved by the Institutional Ethics Board, University of Miami (Miami, USA).

### Behavioral analysis

Analysis of mouse hindlimb locomotor function of α-synuclein knockout and control mice was performed using the Basso Mouse Scale^35^ by investigators blind to genotype. Automated activity boxes (Columbus Instruments) were used to collect total distance traveled and movement time over a 10 min epoch before SCI and at 9d, 12d and 20d post-injury. Because α-synuclein knockout mice are known to have deficits in dopamine signaling, mice were treated with the D1/D5 dopamine agonist SKF-81297 (Cayman Chemicals) to determine if deficient dopamine signaling exacerbated functional deficits after SCI. Preliminary tests comparing four doses of SKF-81297 (0.1, 0.2, 1, 2 mg/kg i.p.) revealed that the 1 mg/kg dose produced the most reliable and robust effects. Therefore, all further experiments used the 1 mg/kg dose. For this analysis, mice received a baseline BMS score on 7d and 14 dpi and then were injected with the dopamine agonist intraperitoneally. Locomotor function was then re-tested 15 min after dopamine agonist treatment by the same raters; again, raters were blinded to genotype.

### Western blot analysis

Naïve (n = 3) and 7d, 21d and 42d post-SCI rats (n = 3–4/group) received a lethal dose of anesthesia (1.5 × surgical dose) and were exsanguinated by transcardial perfusion with PBS. A 5 mm piece of spinal cord centered at the lesion epicenter was rapidly dissected and flash frozen in liquid nitrogen. Protein was isolated with T-PER buffer containing EDTA, phosphatase inhibitors, and protease inhibitors. Homogenized samples were centrifuged for 10 min and 13,000×G at 4 °C and the clear supernatant was removed. Protein concentrations were determined for each sample with the BCA assay (Pierce). 20 µg of protein was separated on a Bis–Tris gel (Invitrogen), transferred to PVDF, blocked with 5% BSA, and then probed for α-synuclein (Cell Signaling D37A6). The intensity of each band was measured with a Kodak imager 4000 mm Pro and levels of α-synuclein were normalized to β-actin control levels.

### Real time PCR analysis

At 21 days post-injury, α-synuclein knockout and control mice (male and female, n = 5–6/group) were given a lethal dose of anesthesia (1.5 × surgery dose) then exsanguinated as above. A 5 mm piece of spinal cord tissue centered on the lesion epicenter was rapidly dissected. Tissue was stored at 4 °C in RNALater (Life Technologies AM7021) until processed for RNA isolation and cDNA generation as previously described^69^. mRNA expression levels of CD68, IL-1β, Iκβα, CXCL1, TNFα, and 18S were examined using a 7900HT Real-Time PCR System (Applied Biosystems). Ribosomal 18S expression was utilized to normalized values for each animal and mRNA levels were calculated using the ΔΔCT method^71^.

### Histological analysis

At 21d post-injury, mice (n = 3–5/group) received a lethal dose an anesthesia (1.5 × surgery dose) and were transcardially perfused with 0.1 M phosphate buffered saline (PBS) until the tissue was cleared of blood. Next, animals were perfused with 100 ml of 4% paraformaldehyde (PFA). The spinal cord was removed and post-fixed in 4% PFA for 2 h followed by phosphate buffer overnight. The next day, the tissue was transferred to 30% sucrose for 3d prior freezing and blocking for tissue sectioning. Spinal cord cross-sections were cut at 10 µm using a cryostat and slide mounted (Superfrost Plus Slides, Fisher Scientific); slides were stored at − 20 °C until used. For tissue analysis, the following targets were visualized using immunohistochemistry: neurofilament-heavy (DSHB, RT97, 1:2,000), GFAP (DAKO, Z0334, 1:1000), NeuN (Abcam 104225 1:10,000), and CD11b (MAC1; Serotec MCA-74G 1:200). Perls staining for iron was performed as previously described^70^ using the Prussian blue stain (Polysciences, Warrington, PA, USA, cat. #24199-1) with DAB intensification.

Axon sparing in mouse tissue was analyzed in cross-sections 600 µm rostral to the injury epicenter immunolabeled for neurofilament. Images of right and left spared white matter were collected at 40 × magnification and with an average position of 150 µm above the central canal and 300 µm lateral to midline. The total number of axons per sample was counted in a standardized 38,206 μm^2^ area using ImageJ and the data for each section averaged.

Macrophage accumulation and neuron sparing in mouse SCI tissue were averaged in tissue sections spanning from 600 µm rostral—caudal to the epicenter using MCID image analysis software. Microglia and macrophages were identified with positive CD11b staining and the proportional area (compared to cross sectional area) was quantified. Neurons were identified by NeuN immunohistochemistry and the total number of positive cells was quantified. Axon number was quantified in sections 600 µm rostral to the epicenter, and GST-π positive oligodendrocytes were measured at the epicenter and 600 µm rostral. White matter sparing was quantified and averaged in sections 600 µm rostral through caudal to the epicenter on tissue stained with eriochrome cyanine and neurofilament. The area of spared white matter divided by cross sectional area was calculated to determine differences between groups.

Human spinal cord samples (Table 1) were deparaffinized in xylene followed by rehydration through ethanol to PBS. For α-synuclein immunohistochemistry, antigen retrieval was performed on the formalin fixed tissue by first incubating sections in citrate buffer (Vector Labs H-3300) at 95 °C for 20 min followed by 20 min to allow the buffer to cool. After the citrate step, the tissue was incubated in 10 µg/ml Proteinase-K for 15 min at 37 °C. The use of citrate buffer and Proteinase-K is an accepted method for staining human tissue with LB509 (Abcam LB509, 1:500)^38^. Tissue sections were then incubated overnight with 1:100 LB509 antibody, which recognizes human α-synuclein from purified Lewy bodies, then processed with secondary antibody, rinsed and coverslipped. For iron detection, Perls Prussian blue stain was used with DAB enhancement. Briefly, sections were deparaffinized as above then rinse with buffer. Endogenous peroxides were quenched with H_2_O_2_ in methanol for 10 min, followed by PBS rinses then incubation in HCl:Potassium Ferrocyanide in a 1:1 ratio. After rinses with buffer, sections were in incubated with DAB + nickel for 5 min, then rinsed and coverslipped.

### Statistical analysis

All histological and behavioral analyses were done in a blinded manner. Statistical analysis was performed using Graph Pad Prism 5.0 (San Diego, California). For analysis of age and gender matching of human controls a two-tailed t-test was used. For analysis of axon and astrocyte profiles in human tissue a Fisher Exact two-tailed test was used. For behavior testing and histological analyses of mouse tissue over distances, two-way repeated measures ANOVA was performed followed by Bonferroni post-hoc analysis to determine group differences. For analysis of within gender neuron counts, a two-tailed t-test was used. Analysis of the number of mice stepping before and after SKF-81297 treatment was performed with a Chi-squared test. Figures were assembled in Photoshop 11.0.2; image manipulation was only performed to increase contrast equally over the entire image when needed so images matched view through microscope.

## Supplementary Information

Supplementary Information.
