# Supplementary material for: Alpha-synuclein increases in rodent and human spinal cord injury and promotes inflammation and tissue loss
Source: Sci Rep. 2021 Jun 3;11:11720. doi: 10.1038/s41598-021-91116-3 (PMC8175699; doi:10.1038/s41598-021-91116-3)
Supplement: Supplementary file 1 — Supplementary Information. [file 41598_2021_91116_MOESM1_ESM.pdf]

Alpha-synuclein increases in rodent and human spinal cord injury and promotes inflammation and tissue loss

Andrew D. Sauerbeck[1], Evan Z. Goldstein[1], Anthony Alfredo[1], Michael Norenberg[2], Alexander Marcillo[2], Dana M. McTigue[1]

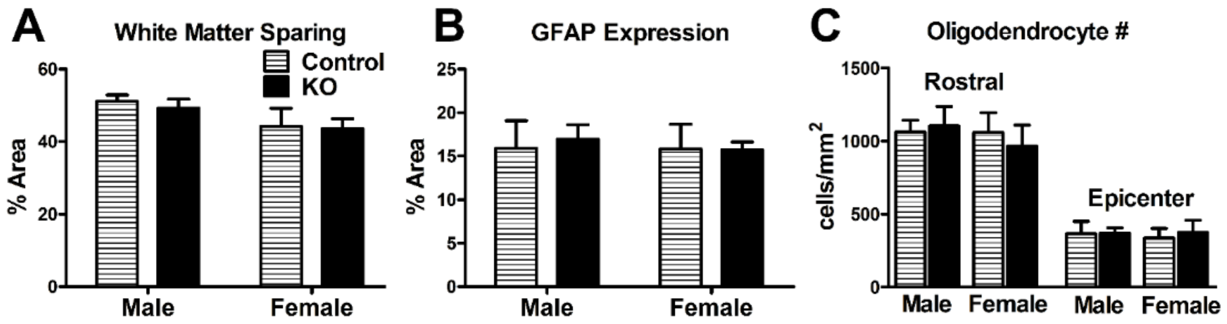

Supplementary Figure 1: α-synuclein knockout did not affect white matter sparing, GFAP expression or the number of oligodendrocytes after spinal cord injury. There was no difference in white matter sparing across the lesion extent (A), GFAP immunoreactivity (B), or the number of GST-π positive oligodendrocytes (C) in KO and control mice.

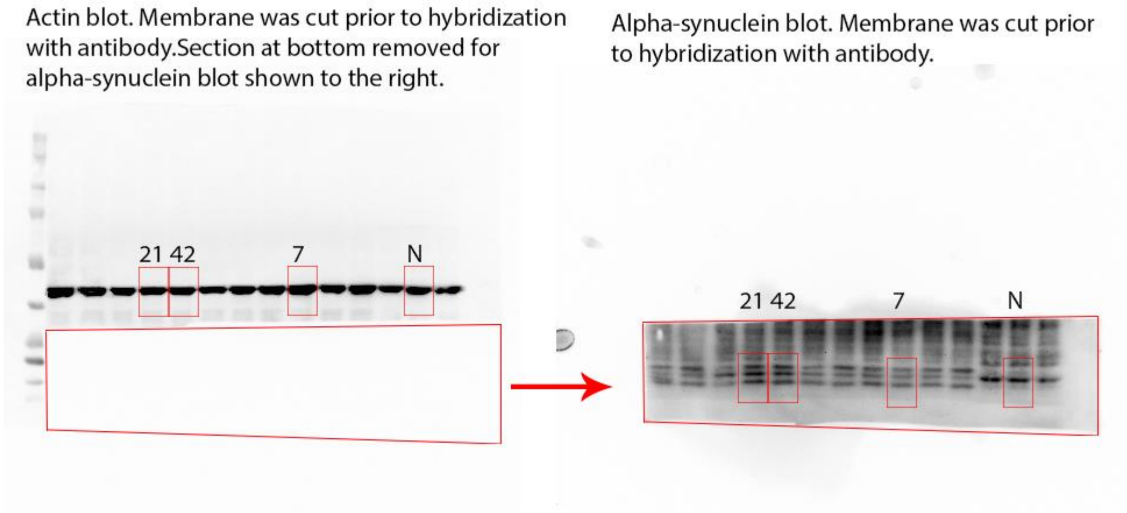

Supplementary Figure 2: Original actin and α-synuclein western blot data utilized in Figure 2.
